# Deficient Letter-Speech Sound Integration Is Associated With Deficits in Reading but Not Spelling

**DOI:** 10.3389/fnhum.2018.00449

**Published:** 2018-11-14

**Authors:** Ferenc Kemény, Melanie Gangl, Chiara Banfi, Sarolta Bakos, Corinna M. Perchtold, Ilona Papousek, Kristina Moll, Karin Landerl

**Affiliations:** ^1^Institute of Psychology, University of Graz, Graz, Austria; ^2^Department of Child and Adolescent Psychiatry, Psychosomatics, and Psychotherapy, Ludwig-Maximilian University, Munich, Germany

**Keywords:** reading deficit, spelling deficit, dyslexia, letter-speech sound integration, cross-modal integration, letter-speech sound interference

## Abstract

Efficient and automatic integration of letters and speech sounds is assumed to enable fluent word recognition and may in turn also underlie the build-up of high-quality orthographic representations, which are relevant for accurate spelling. While previous research showed that developmental dyslexia is associated with deficient letter-speech sound integration, these studies did not differentiate between subcomponents of literacy skills. In order to investigate whether deficient letter-speech sound integration is associated with deficits in reading and/or spelling, three groups of third graders were recruited: (1) children with combined deficits in reading and spelling (RSD, *N* = 10); (2) children with isolated spelling deficit (ISD, *N* = 17); and (3) typically developing children (TD, *N* = 21). We assessed the neural correlates (EEG) of letter-speech sound integration using a Stroop-like interference paradigm: participants had to decide whether two visually presented letters look identical. In case of non-identical letter pairs, conflict items were the same letter in lower and upper case (e.g., “T t”), while non-conflict items were different letters (e.g., “T k”). In terms of behavioral results, each of the three groups exhibited a comparable amount of conflict-related reaction time (RT) increase, which may be a sign for no general inhibitory deficits. Event-related potentials (ERPs), on the other hand, revealed group-based differences: the amplitudes of the centro-parietal conflict slow potential (cSP) were increased for conflicting items in typical readers as well as the ISD group. Preliminary results suggest that this effect was missing for children with RSD. The results suggest that deficits in automatized letter-speech sound associations are associated with reading deficit, but no impairment was observed in spelling deficit.

## Introduction

Strong association between letters and speech sounds is a crucial component of literacy skills. However, knowing letters and corresponding speech-sounds is not sufficient to develop proficient reading; these associations also need to be automatized (letter-sound integration hypothesis, Blomert, 2011). It has been suggested that letter-speech sound integration is deficient in poor readers (Bakos et al., 2017). The current experiment tests the automaticity of letter-speech sound associations with a Stroop-like interference task in 9-year-old children with developmental dyslexia—conceptualized as combined reading and spelling deficit (RSD), a group with isolated spelling deficit (ISD), and a group of typically developing (TD) children. Our aim is to identify whether the automatized nature of letter-speech sound associations is a crucial feature in reading development, or both.

Although reading and spelling skills are generally treated as closely related (Perfetti et al., 1997), the relationship is far from obvious. Some interpret the high correlation between reading and spelling (0.77 < *r* < 0.86) as an indicator that they are two aspects of the same phenomenon (Ehri, 1997). Others highlight that the high correlation between these skills only appears in opaque orthographies, like English, in which letters and speech sounds have various possible mappings (e.g., the “o” is decoded differently in “womb,” “wombat” or “women”). In orthographies with transparent letter-sound correspondences, like German, reading accuracy is close to ceiling and reading fluency is the main criterion to measure reading skills. In these languages, reading fluency and spelling skills only show a moderate correlation (e.g., Moll and Landerl, 2009).

In accordance with only moderate correlations, dissociations have been reported between impairments in spelling and reading fluency (Wimmer and Mayringer, 2002; Moll and Landerl, 2009; Moll et al., 2014). Developmental studies showed that the prevalence of isolated, as well as combined RSDs (the latter usually referred to as dyslexia) are around 6%–8% in German (Moll and Landerl, 2009), while the combined deficit has a somewhat higher prevalence in French (Fayol et al., 2009). Neuropsychological studies also reported a double dissociation of reading and spelling skills (De Renzi et al., 1987; Mochizuki and Ohtomo, 1988). These studies argue that the underlying core problems are different. Isolated reading fluency deficit may be a consequence of impaired access to orthographic representations, while these representations are available for top-down spelling processes (Moll and Landerl, 2009). ISD, on the other hand, may be the result of a reduced orthographic lexicon. Reading skills are not affected, as even the reduced orthographic lexicon is sufficient for word recognition, i.e., reading (Frith, 1980). Another explanation of the lack of reading problems in individuals with ISD suggests that they may use highly efficient decoding strategies, which compensate for the deficient orthographic knowledge (Moll and Landerl, 2009). The dissociation between RSDs is further supported by evidence showing different cognitive profiles associated with impairments in reading fluency and spelling (Wimmer and Mayringer, 2002). Thus, spelling problems have been associated with phonological deficits, whereas reading fluency problems with difficulties in rapid automatized naming (i.e., the serial naming of repeated items presented in lines or columns), which is an indicator of visual-verbal access.

The distinct core deficits in reading vs. spelling impairment also suggest different patterns in automatized letter-speech sound associations. That is, since children with a spelling deficit have a reduced orthographic lexicon (Frith, 1980) but efficient decoding strategies (Moll and Landerl, 2009), they are expected to demonstrate preserved letter-speech sound associations. On the other hand, children with combined RSDs (i.e., dyslexia) are expected to show atypical automatized letter-speech sound associations, due to deficient access to orthographic representations. Thus, atypical automated letter-speech sound associations should be related to reading, and not spelling impairment.

The following section reviews previous results of letter-speech sound associations in dyslexia, and describes a classical method (Posner and Mitchell, 1967) that has been applied to assess letter-speech sound associations using event-related potential (ERP) in a novel study (Bakos et al., 2017). Since the current study is an ERP study, the introduction follows with the description of conflict-related ERP components, and then turns to the description of the current study.

## Letter-Speech Sound Associations in Dyslexia

Previous studies mainly used a passive oddball mismatch negativity (MMN) method to assess the neural correlates of crossmodal letter-speech sound associations. MMNs are elicited by deviant stimuli (Näätänen et al., 1978, 1993), and are interpreted as correlates of memory functions, violation detection or predictive functions (for a review, see Winkler, 2007). Although the MMN methodology mainly uses unimodal auditory or visual stimuli (Czigler, 2007), the method was also adapted to crossmodal associations, like letter-speech sound correspondences (Froyen et al., 2008; Moll et al., 2016). In crossmodal adaptations, visual (letter) and auditory (speech sound) stimuli were presented simultaneously. Auditory (Froyen et al., 2008) but not visual (Froyen et al., 2010) MMNs were boosted by simultaneous congruent information, but only in advanced and not beginning readers (Froyen et al., 2009; Jones et al., 2016) and also not in children with dyslexia (Froyen et al., 2011). These results have been integrated, suggesting that automatization of letter-speech sound associations develops with reading proficiency, but not in the case of a reading deficit (Blomert, 2011). Results further suggest that the crossmodal MMN deficit is most pronounced when the auditory and visual stimuli come simultaneously (Žarić et al., 2014). On the other hand, the impairment was found to be at least partially reversible (Žarić et al., 2015). Reading skills also correlated with the elicited MMN measures of letter-speech sound integration (Žarić et al., 2014, 2015). Others, however, did not replicate absent crossmodal MMN effect in dyslexia, but observed a delay (Moll et al., 2016).

While the MMN methodology has been successfully adapted to crossmodal events, a disadvantage of the method is rooted in its passive nature. Long passive observation tasks are difficult to administer with school aged children. In addition, behavioral data that allows controlling performance rate and attention are not available in passive tasks. Using an active priming task, Nash et al. (2017) found that children with dyslexia show a pattern similar to a reading-age-matched control group, which suggests that letter-speech sound integration is a function of reading proficiency.

Similar results were borne out by Bakos et al. (2017), using an adapted Stroop-like letter-speech sound interference paradigm (Posner and Mitchell, 1967). Throughout the task, participants saw two letters, and had to decide whether the two letters are visually identical or not by pressing a response key. The letter pairs could be the same letter with the same visual features (e.g., “t t” or “T T”: “yes” answer), different letters (e.g., “T k” or “t K”: “no” answer), or the same letter in different cases (e.g., “T t”: “no” answer). The critical comparison is between the two “no” answer conditions, which differ in whether they are conflicting or not. Conflict emerges from the same letters presented in different cases (e.g., “T t”): they are visually different but are associated with the same phoneme. A novel ERP study used this task to compare neural correlates of conflict in RSD and TD children, and found a similar reaction time (RT) increase to conflict trials in both groups, but conflict-related ERP amplitude modulation was missing in RSD (Bakos et al., 2017).

The current study replicates and extends the Bakos et al. (2017) study by using the same method but contrasting the effect of reading vs. spelling deficit on the automatization of letter-speech sound associations. Since the method is based on interference processing, it is important to differentiate between general inhibitory processes and processes related to letter-speech sound integration and how they are associated with dyslexia. Although a number of articles found atypical inhibitory performance in dyslexia (Everatt et al., 1997; Helland and Asbjørnsen, 2000), it is not clear, whether the deficit is rooted in the overall higher response latencies of dyslexic children (Das, 1993; Protopapas et al., 2007; Faccioli et al., 2008), or in the fact that some experimental tasks loaded on reading skills or employed letter-based stimuli (Reiter et al., 2005; Bakos et al., 2017). To avoid confounding effects, the current study tests both behavioral and ERP measures of conflict processing. The following section provides an overview of conflict-related ERP components, and their realization in children with dyslexia.

## Conflict-Related ERP Components

Previous studies addressed conflict processing and conflict resolution mainly with Stroop or Flanker tasks. These studies identified three crucial components of conflict identification, conflict monitoring and conflict resolution: an N1 (Yu et al., 2015) and an N2 component (Larson et al., 2014), as well as a late positive complex (West, 2003), respectively.

The N1 is a negative fronto-central component peaking between 100 ms and 200 ms. While the N1 was shown to be sensitive to conflict detection (Yu et al., 2015), both conflict-related amplitude increase (Johnstone et al., 2009) as well as decrease (Yu et al., 2015) have been reported. Previous studies have also found atypical N1 amplitude modulation in adults (Mahé et al., 2014), and children with dyslexia (Bakos et al., 2017).

The second, N2 component peaks between 250 ms and 350 ms, and has a maximum over fronto-central electrodes. Previous studies argue that this component results from conflict detection and monitoring, originating from the anterior cingulate cortex (Larson et al., 2009, 2014). Similar to the N1 component, some results showed decreased (Yu et al., 2015), while others reported increased amplitudes for conflict vs. non-conflict trials (Johnstone et al., 2009). Yet others found no conflict-related N2 amplitude modulation either in TD children, or in children with dyslexia (Henkin et al., 2010; Bakos et al., 2017). A further adult study observed altered N2 amplitude modulations for conflict in the flanker task, but only in typical readers, not in individuals with dyslexia (Mahé et al., 2014).

The last conflict-related component is the conflict slow potential (cSP), which is a late positive complex. The complex begins approximately 500 ms after stimulus onset and is observable over the centro-parietal electrodes. The cSP has been hypothesized to originate from lateral and posterior cortices (West, 2003; Hanslmayr et al., 2008; Larson et al., 2014), and to reflect conflict resolution (West, 2003) or response selection (West et al., 2005). Bakos et al. (2017) found a marginally significant amplitude decrease to conflict trials in TD children, but not in children with dyslexia.

## The Current Study

The current study is a replication and extension of the Bakos et al. (2017) study. As described above, Bakos et al. (2017) tested typical readers as well as children with combined RSDs in a letter-speech sound interference task and found a significant conflict-related N1 as well as cSP amplitude decrease in typical development. Both effects were missing in children with combined RSDs. Neither group showed any signs of N2 conflict-sensitivity.

We are aimed at replicating the results, expecting that children with dyslexia (combined RSD) show deficient conflict processing with stimuli relying on automatized letter-speech sound associations. The deficit is expected to root in the deficient access to the orthographic representations. Extending the previous design, we also tested children with ISD. ISD is associated with reduced orthographic lexicon, which does not affect reading abilities, due to the use of underspecified orthographic representations for reading (Frith, 1980) and/or efficient decoding skills (Moll and Landerl, 2009). Thus, we expect typical automated letter-speech sound associations in ISD. We also expect that none of the groups show an impairment in general inhibitory measures, as revealed by a comparable increase in RTs for the conflicting trials. To this end, we expect increased RTs in conflict processing in all three groups.

## Materials and Methods

### Participants

Altogether 48 children participated in the study. Children were selected based on a screening of 3rd graders in primary schools in and around Graz, Austria. Initially, reading (Wimmer and Mayringer, 2014) and spelling skills (Müller, 2004) were assessed in a classroom setting. Later, reading skills were reassessed using individual 1-min word and pseudoword reading tasks (Moll and Landerl, 2010). Three groups were selected based on the screening: children with combined RSD, children with ISD and TD children. RSD children had both reading and spelling skills ≤20th percentile. Children with ISD had spelling skills ≤20th percentile, but ≥25th percentile in reading. TD children performed ≥25th percentile on both reading and spelling. All children were monolingual German speakers, had an IQ ≥85 (Weiß, 2006), and had normal or corrected vision. No children had a history of sensory or neurological deficits, had a clinical diagnosis of ADHD, or an above-threshold score on a parental questionnaire for attention deficits (FBB-ADHS, DISYPS-II, Döpfner et al., 2008). The final pool of participants was composed of 10 children with RSD, 17 children with ISD and 21 TD children. Age, IQ, reading and spelling abilities for each of the groups are provided in Table 1.

**Table 1:** Descriptive statistics of participants.

	RSD (*N* = 10)	ISD (*N* = 17)	TD (*N* = 21)	Group comparisons
**Age**				
Mean (SD)	9.36 (0.33)	9.75 (0.68)	9.49 (0.35)	
Min-Max	8.75–9.67	8.75–11.0	8.83–10.33	
**IQ^1^**				
Mean (SD)	96.80 (9.08)	99.53 (8.12)	103.71 (9.67)	
Min-Max	87–119	85–115	91–121	
**Reading speed^2^**				
Mean (SD)	10.00 (10.81)	56.12 (14.70)	53.43 (17.57)	RSD < ISD = TD
Min-Max	1–34	34–86	28–89	
**Word reading^3^**				
Mean (SD)	8.10 (6.62)	51.00 (19.32)	46.52 (13.49)	RSD < ISD = TD
Min-Max	1–18	27–87	27–67	
**Pseudoword reading^4^**				
Mean (SD)	11.90 (8.85)	50.29 (25.69)	54.67 (14.05)	RSD < ISD = TD
Min-Max	2–31	19–95	34–71	
**Spelling^5^**				
Mean (SD)	9.10 (6.97)	11.47 (4.78)	46.81 (11.65)	RSD = ISD < TD
Min-Max	0–17	5–20	28–68	

*Note. 1: CFT-IQ (Weiß, 2006), 2: percentiles of reading speed on SLS 2–9 (Wimmer and Mayringer, 2014), 3: percentiles of 1-min word reading and 4: 1-min pseudoword reading on SLRT-II (Moll and Landerl, 2010), 5: percentiles of spelling on DRT 3 (Müller, 2004). For all group comparisons, p < 0.001*.

EEG recording took place in an acoustically and electrically shielded examination room at the University of Graz. An examiner stayed with the children throughout the testing session to provide support and monitor adherence to the testing protocol. Children received 25 € for their participation. This study was carried out in accordance with the recommendations of the Ethical Committee of the University of Graz. The protocol was approved by the University of Graz. Parents of all subjects gave written informed consent in accordance with the Declaration of Helsinki.

### Stimuli and Procedure

Children were shown two letters at the same time and were instructed to press a certain key on the keyboard when the two letters were visually identical (i.e., “looked the same”) and another response key when the letters were visually different (i.e., “looked different”). Identical pairs were upper case or lower case (e.g., “T T” or “k k”), different pairs were different letters (Non-conflict items, e.g., “T k” or “t K,” one of the letters always lower case, the other always upper case), or the same letter in upper case and lower case (Conflict items, e.g., “T t”). There were 45 lower case and 45 upper case Same items, 60 Conflict and 60 Non-conflict different items. Participants were instructed to respond as fast as possible by pressing “p” for same, and “q” for different items on a QWERTZ keyboard^1^.

The items were composed of a crosshair shown for 1,000 ms, then the letter pair appearing in 57-pt Arial in the middle of the screen until response. The response was followed by a blank screen for 1,000 ms. Altogether, 210 items were used in two blocks of 105 items. The order of the stimuli was randomized, and participants had a self-paced break between the blocks.

### EEG Recording and Preprocessing

EEG recording was done from 19 channels according to the international 10-20 system, using a Brainvision BrainAmp Research Amplifier (Brain Products, sampling rate of 500 Hz, resolution 0.1 μV) and a stretchable electrode cap, referenced to the nose and re-referenced offline to a mathematically averaged ears reference (Essl and Rappelsberger, 1998; Hagemann, 2004; Papousek et al., 2016). Impedance was kept below 5 kΩ. EOG measures were obtained to identify ocular artifacts. The vertical EOG was recorded from the supra- and sub-orbit of the right eye, the horizontal EOG was recorded from the outer canthi using adhesive Ag/AgCl electrodes. The continuous EEG was filtered (low cutoff: 0.1 Hz, time constant: 15.91, 24 dB/Oct; high cut off: 100 Hz, 24 dB/Oct; notch filter: 50 Hz), EOG artifacts were removed by automatic ocular correction, using an ICA algorithm as implemented in BrainVision Analyzer 2.0 (slope mean, over the whole data, ICA with infomax algorithm, total squared correlations to delete: 30%; Gratton et al., 1983). Then data was segmented into epochs of −100 to 700, in which the time window of −100 to 0 served as the basis for baseline correction. Only segments with a correct response outside the 0–700 ms time window were considered. Other artifacts were excluded automatically (gradient criteria: more than 50 μV difference between two successive data points or more than 200 μV difference in a 200 ms window; absolute amplitude criteria: amplitudes exceeding +100 or −100 μV; low activity criterion: less than 0.5 μV activity in a 100 ms window). All participants had at least 26 valid segments in each of the two “Different” conditions, thus all children were included in the analyses. The mean number of included conflict and non-conflict epochs were 53.20 (SD = 8.59) and 51.40 (SD = 8.45) for the RSD, 50.41 (8.19) and 49.35 (9.50) for the ISD and 52.10 (6.50) and 52.38 (6.99) for the TD group.

**Figure 1:**
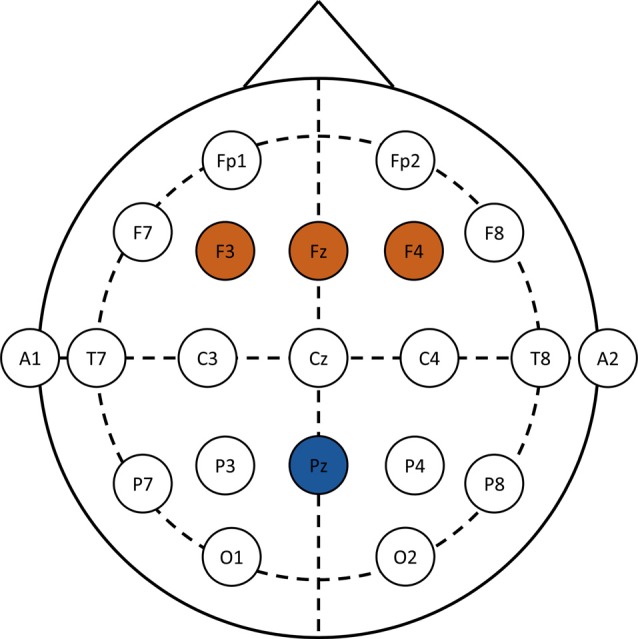
EEG montage and regions of interest. N1 and N2 components are analyzed over F3, Fz and F4 (brown), whereas the conflict slow potential (cSP) data is analyzed over the Pz (blue) electrode.

F3, Fz and F4 electrodes were pooled for the analyses of the N1 and N2 components. The time window for the N1 components was between 90–170 ms after stimulus onset, whereas the time window for the N2 component was between 310–380 ms. For the cSP, the Pz electrode was considered between 500 ms and 700 ms after the onset of the stimulus. Regions of interest and time windows were based on a previous studies using the same paradigm (Bakos et al., 2017). EEG montage and regions of interest are provided in Figure 1.

## Results

### Behavioral Measures: Accuracy and Reaction Times

**Figure 2:**
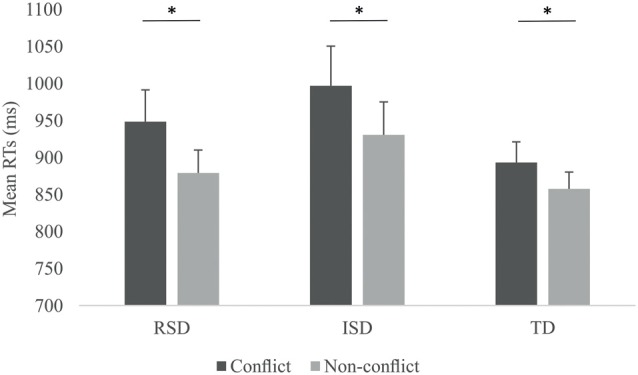
Reaction times (RTs) by Stimulus-type and by Group. Error bars indicate SEM. Asterisks indicate significant differences (*p* < 0.05).

First behavioral measures were analyzed. Since accuracies for the two stimulus-type conditions across the three groups were above 97.5%, accuracies were not analyzed directly. In the case of RTs, only RTs for correct answers were considered. We calculated the median RTs for Conflict as well as Non-conflict items for all participants. To account for speed-accuracy trade-off, RTs were corrected by dividing them with the corresponding accuracy. RTs by Stimulus-type and by Group are provided on Figure 2. We ran a 2 × 3 mixed ANOVA with Stimulus-type (Conflict vs. Non-conflict) as within-subject and Group (RSD vs. ISD vs. TD) as between subject variable. The ANOVA revealed a significant main effect of Stimulus-type, *F*_(1,45)_ = 33.002, *p* < 0.001, $\eta_{\text{p}}^{2}$ = 0.423, with higher RTs for Conflict than for Non-conflict items. Neither the main effect of Group, nor the Stimulus-type × Group interaction were significant (both *p*s > 0.278). To confirm that all three groups indeed showed significantly higher RTs for conflict than for non-conflict items, we ran separate repeated measures ANOVAs for each group with Stimulus-type as within-subject variable. A significant effect of stimulus-type was confirmed for all three groups, *F*_(1,9)_ = 15.838, *p* = 0.003, $\eta_{\text{p}}^{2}$ = 0.638 for the RSD group, *F*_(1,16)_ = 22.382, *p* < 0.001, $\eta_{\text{p}}^{2}$ = 0.583 for the ISD group and *F*_(1,20)_ = 4.842, *p* = 0.040, $\eta_{\text{p}}^{2}$ = 0.195 for the TD group.

### Early Frontal Correlates of Conflict—N1 and N2

**Figure 3:**
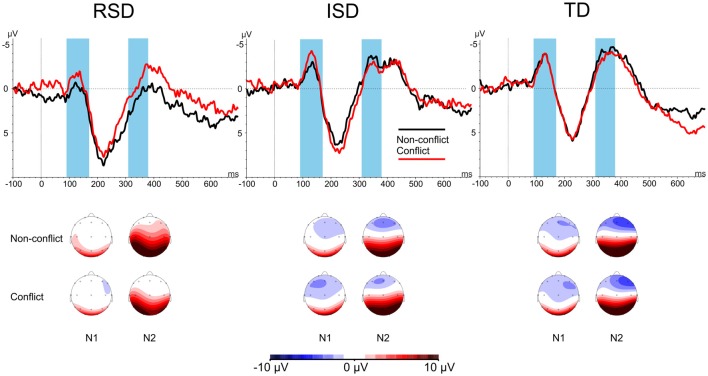
N1 and N2 amplitudes by Stimulus-type and by Group. Highlighted areas indicate the N1 (90–170 ms after stimulus onset) and N2 (310–380 ms after stimulus onset) time windows. Scalp maps show the averaged activity in the highlighted time windows.

Next, conflict-related N1 peak amplitudes were analyzed. In accordance with previous studies (Moll et al., 2016; Bakos et al., 2017) pooled signals from the F3, Fz and F4 electrodes were used with Stimulus-type (Conflict vs. Non-conflict) as within-subject and Group (RSD vs. ISD vs. TD) as between subject variables. The same analysis was carried out for both the N1 and N2 components. N1 and N2 amplitudes by Stimulus-type and Group are provided in Figure 3. For N1, the 2 × 3 mixed ANOVA revealed a significant main effect of Group, *F*_(2,45)_ = 3.411, *p* = 0.042, $\eta_{\text{p}}^{2}$ = 0.132. No other effects were significant (all *p*s ≥ 0.236). Since no conflict related effect were found, no further analyses were conducted.

Similarly, the ANOVA for N2 peak amplitudes showed only a significant main effect of Group, *F*_(2,45)_ = 3.774, *p* = 0.031, $\eta_{\text{p}}^{2}$ = 0.144. No other effects were significant, all *p*s ≥ 0.266.

### Late Parietal Correlates of Conflict—cSP

**Figure 4:**
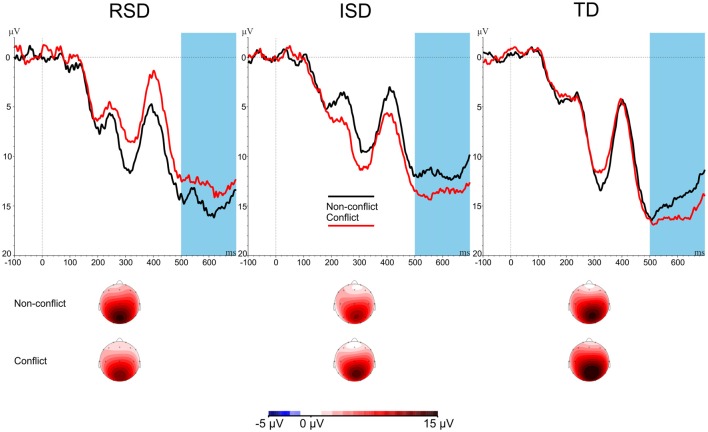
cSP amplitudes by Stimulus-type and by Group. The highlighted area indicates the cSP time window (500–700 ms after stimulus onset). Scalp maps show the averaged activity in the highlighted time windows.

Next, conflict-related slow potentials were analyzed over the centro-parietal Pz electrode. Conflict-related amplitudes are provided in Figure 4. A 2 × 3 mixed ANOVA was used with Stimulus-type (Conflict vs. Non-conflict) as within-subject variable, and Group (RSD vs. ISD vs. TD) as between subject variable. The ANOVA revealed a significant Stimulus-type × Group interaction, *F*_(2,45)_ = 3.284, *p* = 0.047, $\eta_{\text{p}}^{2}$ = 0.127. No other effects were significant, all *p*s ≥ 0.290. Amplitudes for cSP are provided on Figure 4.

To further analyze the Stimulus-type × Group interaction, a separate repeated-measures ANOVA was conducted for each group with Stimulus-type (Conflict vs. Non-conflict) as within-subject variable. The ANOVAs revealed a significant effect for the TD group, *F*_(1,20)_ = 6.801, *p* = 0.017, $\eta_{\text{p}}^{2}$ = 0.254, as well as for the ISD group, *F*_(1,16)_ = 4.946, *p* = 0.041, $\eta_{\text{p}}^{2}$ = 0.236. For the TD and ISD groups, amplitudes for conflict were more positive than for non-conflict items. The RSD group showed the reverse pattern, this difference, however, was not significant, *F*_(1,9)_ = 0.907, *p* = 0.366, $\eta_{\text{p}}^{2}$ = 0.092.

## Discussion

The current study tested the automaticity of letter-speech sound associations using an interference task. Three groups of 3rd graders were tested: a group with combined RSD, a group with ISD, and a group of typical readers (TD). Results showed no general inhibitory deficit, as all three groups showed a comparable RT increase for conflicting trials. However, the neural signatures of conflict processing differed between the groups. Whereas the N1 and N2 components remained unaffected, cSP amplitudes were modulated differently throughout the groups. In the case of the TD and ISD groups, conflicting events elicited a more positive cSP than non-conflicting events. The RSD group on the other hand showed a non-significant reverse pattern. That is, the lack of the effect is not a power issue, but the consequence of different conflict processing. Note, however, that there were only 10 participants in the RSD group. Further supporting analyses are provided in Supplementary Data Sheet 1 (Supplementary Analysis 1). These analyses compare the 10 RSD participants to 10 ISD children matched on spelling skills, and 10 TD children with reading skill matching the selected ISD participants.

To sum up, the most important results are that all groups show a comparable behavioral difference between non-conflict and conflict items, suggesting that general inhibition may not be deficient in any of the groups. ERP results, however, show typical patterns of conflict-processing in the ISD group, but no effect in the RSD group. That is, letter-speech sound integration deficits are more closely associated with reading skills, whereas no similar effects were observed for spelling.

Based on the findings, the discussion focuses on three relevant issues: (1) how can the lack of a deficit in letter-speech sound integration in ISD be explained in terms of previous theoretical frameworks; (2) how can the current results of the RSD group be reconciled with Bakos et al.’s (2017) notion that deficient automatized letter-speech sound associations are related to reading impairment; and (3) how can the relation between cSP amplitude modulation and activation of phonological information be explained.

First, the typical conflict-related amplitude modulation in ISD might be associated with decoding skills. Previous theoretical frameworks by Moll and Landerl (2009) suggest that children with ISD have a reduced orthographic lexicon, which is compensated by highly efficient decoding strategies. The current results are in line with this hypothesis, as typical letter-speech sound associations were observed in ISD.

The current results are also in line with previous studies showing that sensitivity to letters is a crucial factor predicting reading performance in TD children (Kemény et al., 2018). Our findings corroborate earlier evidence that individuals with reading deficit may be impaired in print sensitivity (Maurer et al., 2006, 2011; Hasko et al., 2013; Fraga González et al., 2014; Araújo et al., 2015), but this impairment might only appear from a certain age onwards (for discussion, see Kemény et al., 2018). Since the cited evidence does not stem from crossmodal processing, it is a question whether the core deficit is in fact associated to letter-speech sound associations, or rather to letter-based effects. The current study was not designed to address this question though.

### Replication Differences

The current study replicated the findings of Bakos et al. (2017) in a number of ways: both studies found RT effects of stimulus-type in all groups and interpreted those as a sign of intact general inhibitory mechanisms. Both studies found atypical conflict processing in RSD, although the temporal windows were different: Bakos et al. (2017) reported differences on the N1 as well as the cSP amplitudes, whereas the current study only found differences in the cSP amplitude modulation (see below).

RTs of the Bakos et al. (2017) study were also higher: TD children responded on average in 1,059 ms to Non-conflict and 1,097 ms to Conflict trials, and the measures were 1,167 ms to Non-conflict and 1,185 ms to Conflict trials in children with dyslexia. The RTs observed in the current study were 857 and 893 ms for TD and 879 and 948 ms for the RSD group. This is a difference around 200 ms (22%) in TD, and 260 ms (29%) in the RSD group.

As the selection criteria were identical, differences might be rooted in the sample characteristics. As shown in Table 2, between group differences on both word reading and spelling skills were smaller in our sample than in the sample of Bakos et al. (2017). Group-differences in word reading skills may be crucial for the integration of the results. Both the Bakos et al. (2017) and the current results argue that conflict-related amplitude modulations are associated with reading abilities, with only good readers exhibiting conflict sensitivity. Reading abilities of TD children in Bakos et al. (2017), though, were better than in the current study (difference is more than 7.5 percentile). Thus, it is plausible, that the differences in the N1 amplitude modulation are rooted in reading skills: better readers show earlier sensitivity, less good readers only show later effects (cSP), and children with reading deficit show no effect at all. The current study was not planned for such an analysis, an individual differences design with a larger sample size could provide further insights.

**Table 2:** Comparing participant characteristics of the current experiment, and the Bakos et al. (2017) study.

	TD group		RSD group		TD-Bakos et al.		RSD-Bakos et al.	
	(*n* = 21)		(*n* = 10)		(*n* = 37)		(*n* = 36)	
	M	SD	M	SD	M	SD	M	SD
Age	9.49	0.35	9.36	0.3	9.47	0.32	9.5	0.5
IQ^1^	103.71	9.68	96.80	9.08	110.57	10.59	109.14	13.38
Reading speed^2^	53.43	17.57	10.00	10.81	52.05	12.85	10.19	8.75
Word reading^3^	46.52	13.49	8.10	6.62	54.15	17.35	7.28	6.4
Pseudoword reading^4^	54.67	14.05	11.90	8.85	53.6	19.57	12.17	8.58
Spelling^5^	46.81	11.65	9.10	6.97	57.46	11.82	9.94	6.16

*Note. 1: CFT-IQ (Weiß, 2006), 2: percentiles of reading speed on SLS 2–9 (Wimmer and Mayringer, 2014), 3: percentiles of 1-min word reading and 4: 1-min pseudoword reading on SLRT-II (Moll and Landerl, 2010), 5: percentiles of spelling on DRT 3 (Müller, 2004)*.

### Activation of the Phonological Codes

Our results replicate those of Bakos et al. (2017) showing no conflict-related amplitude modulations of the cSP in dyslexia. Bakos et al. (2017) provided two possible explanations to this phenomenon that could not be distinguished based on their results: on the one hand, phonological codes might not be activated in developmental dyslexia until 900 ms after stimulus onset. This was a reasonable assumption, provided that the mean RT of RSD children was 1,167 ms to Non-conflict and 1,185 to Conflict items, whereas the cSP was only analyzed until 900 ms. It is thus possible, that the typical pattern emerges between 900 ms and response. On the other hand, *automatic* phonological activation might not take place at all in children with developmental dyslexia.

In the current study, we found no conflict related cSP amplitude modulation in the 500–700 ms time window. Supplementary Data Sheet 2 (Supplementary Analysis 2) provides an analysis of the cSP in the −200 to 0 ms time window before response, where we still found no conflict-related amplitude modulation in the RSD group, arguing against late activation. That is, such an experimental design may not elicit *automatic* activation of the phonological codes in RSD. While behavioral results (that is, RT increase to conflicting trials) may not differentiate between automatic and controlled processes, cSP amplitudes may only be sensitive to the former, not the latter. This hypothesis should be supported by focused ERP experiments contrasting automatic and controlled processes.

## Conclusion

The current study was aimed at testing whether automatized letter-speech sound associations contribute to reading skills, spelling skills or both. We tested children with combined RSD, children with ISD, and TD children. We used a letter-speech sound interference task, in which conflict was evoked by presenting the same letter in different cases (but representing the same phoneme). The current study argues that neither reading deficit, nor spelling deficit is associated with impaired inhibition. At the same time, typical conflict-related N1, N2 and cSP amplitude modulations are not observed in RSD. Results argue, that automatized letter-speech sound association is a crucial factor in the development of reading fluency skills (Blomert, 2011).

## Author Contributions

KL and KM were involved in the conceptualization and design of the study. SB, IP and KM developed and applied the methodology. FK, MG, CB and CP acquired the data. FK performed the analyses and wrote the manuscript.

## Conflict of Interest Statement

The authors declare that the research was conducted in the absence of any commercial or financial relationships that could be construed as a potential conflict of interest.

## Footnotes

^1^All other keys were removed from the keyboard.
