# Supplementary material for: Deficient Letter-Speech Sound Integration Is Associated With Deficits in Reading but Not Spelling
Source: Front Hum Neurosci. 2018 Nov 14;12:449. doi: 10.3389/fnhum.2018.00449 (PMC6246711; doi:10.3389/fnhum.2018.00449)

Deficient letter-speech sound integration is associated with deficits in reading but not spelling

Supplementary analysis 1.

Ferenc Kemény^1^, Melanie Gangl^1^, Chiara Banfi^1^, Sarolta Bakos^2^, Corinna M. Perchtold^1^, Ilona Papousek^1^, Kristina Moll^2^, Karin Landerl^1^

^1^ Institute of Psychology, University of Graz, Austria

^2^ Department of Child and Adolescent Psychiatry, Psychosomatics, and Psychotherapy, Ludwig-Maximilian University, Munich, Germany

In a further analysis, we matched participants of the three groups on reading and spelling skills. 10 ISD children were selected to match on spelling skills with the 10 RSD skills. 10 TD children were selected to match the 10 selected ISD children on their word reading skills. Supplementary Table 1 provides the descriptive statistics of these 30 children by group. Conflict-related cSP amplitude modulation was compared across groups.

A 2 (Stimulus-type) x 3 (Group) ANOVA revealed a significant interaction, F(2,27) = 3.779, p = 0.036, η_p_^2^ = 0.219. Neither the stimulus-type, nor the group main effect were significant (both ps > 0.129).

Separate analysis of the groups revealed no stimulus-based effect for the RSD group, F(1,9) = 0.907, p = 0.366, η_p_^2^ = 0.092 (same as the result shown in the paper). Both other groups showed a significant stimulus-based effect, F(1,9) = 7.738, p = 0.021, η_p_^2^ = 0.462 for the ISD and F(1,9) = 6.233, p = 0.034, η_p_^2^ = 0.409. Supplementary Figure 1 illustrates the cSP amplitudes on the Pz electrode.

| Supplementary Table 1. | | | | |  |
| --- | --- | --- | --- | --- | --- |
| Descriptive statistics of participants. | | | | | |
|  |  | RSD (N=10) | ISD (N=17) | TD (N=21) | Group comparisons |
| Age | | | | |  |
|  | mean (SD) | 9.36 (0.33) | 9.77 (0.81) | 9.52 (0.42) |  |
|  | min-max | 8.75-9.67 | 8.75-11.00 | 9.00-10.33 |  |
| IQ^1^ |  |  |  |  |  |
|  | mean (SD) | 96.80 (9.08) | 98.10 (8.91) | 102.50 (9.26) |  |
|  | min-max | 87-119 | 85-112 | 91-115 |  |
| Reading speed^2^ | | | | |  |
|  | mean (SD) | 10.00 (10.81) | 55.50 (14.30) | 53.10 (17.43) | RSD < ISD = TD |
|  | min-max | 1-34 | 34-71 | 31-89 |  |
| Word reading^3^ | | | | |  |
|  | mean (SD) | 8.10 (6.62) | 45.00 (13.41) | 44.20 (12.91) | RSD < ISD = TD |
|  | min-max | 1-18 | 30-70 | 29-67 |  |
| Pseudoword reading^4^ | | | | |  |
|  | mean (SD) | 11.90 (8.85) | 47.00 (25.41) | 52.00 (13.42) | RSD < ISD = TD |
|  | min-max | 2-31 | 19-87 | 35-71 |  |
| Spelling^5^ | | | | |  |
|  | mean (SD) | 9.10 (6.97) | 10.90 (4.82) | 46.30 (11.44) | RSD = ISD < TD |
|  | min-max | 0-17 | 5-17 | 31-68 |  |
| ***Note***. 1: CFT-IQ (Weiß, 2006), 2: percentiles of reading speed on SLS 2-9 (Wimmer & Mayringer, 2014), 3: percentiles of one-minute word reading and 4: one-minute pseudoword reading on SLRT-II (Moll & Landerl, 2010), 5: percentiles of spelling on DRT 3 (Müller, 2004). For all group comparisons, p < 0.001. | | | | | |

**Supplementary Figure 1**. cSP amplitudes by Stimulus-type and by Group (with 10 participants in each group). The highlighted area indicates the cSP time window (500-700 ms after stimulus onset).


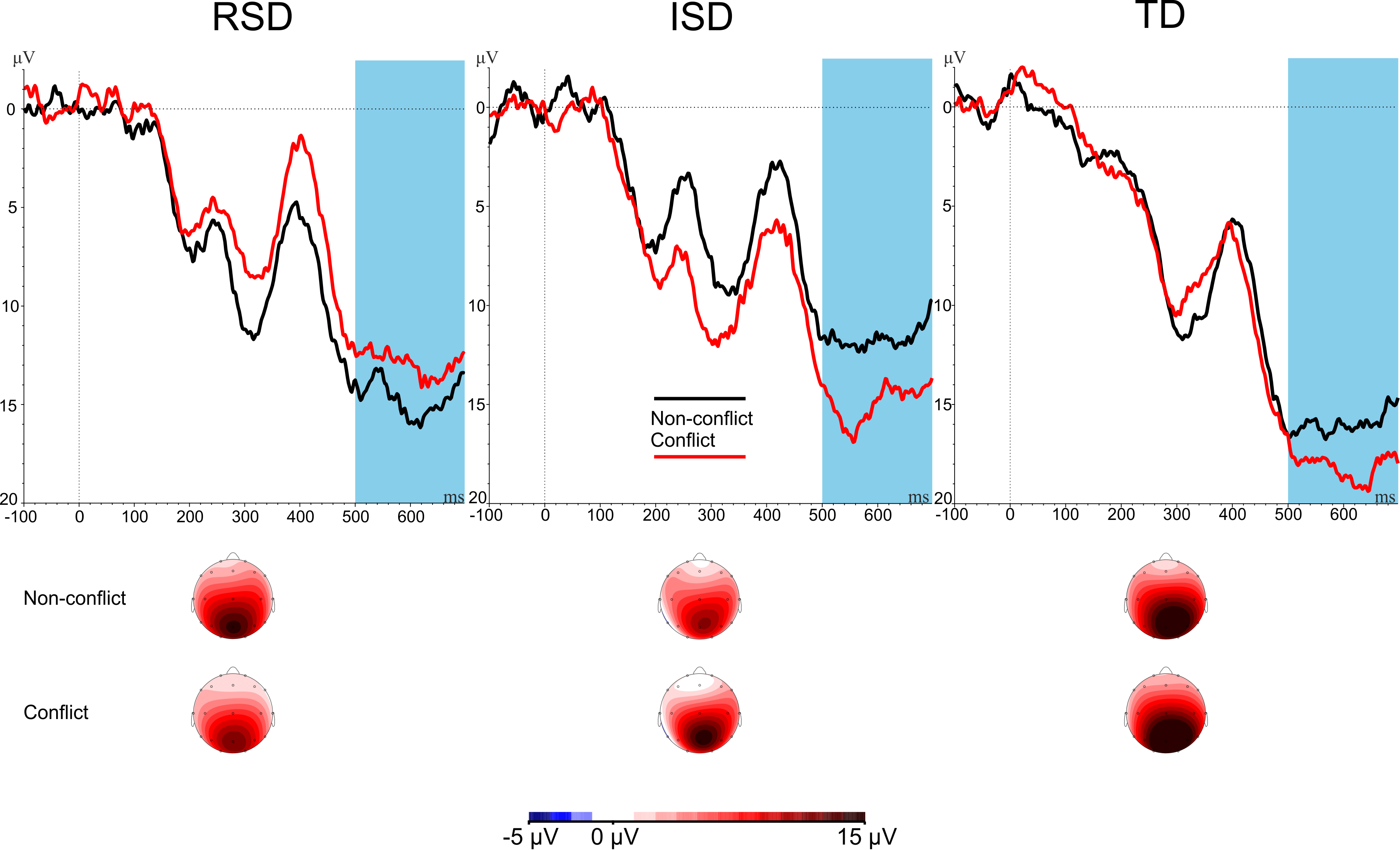

Supplement: Supplementary file 1 [file Data_Sheet_1.docx]
