# Supplementary material for: Deficient Letter-Speech Sound Integration Is Associated With Deficits in Reading but Not Spelling
Source: Front Hum Neurosci. 2018 Nov 14;12:449. doi: 10.3389/fnhum.2018.00449 (PMC6246711; doi:10.3389/fnhum.2018.00449)
Supplement: Supplementary file 2 [file Data_Sheet_2.docx]

Deficient letter-speech sound integration is associated with deficits in reading but not spelling

Supplementary analysis 2.

Ferenc Kemény^1^, Melanie Gangl^1^, Chiara Banfi^1^, Sarolta Bakos^2^, Corinna M. Perchtold^1^, Ilona Papousek^1^, Kristina Moll^2^, Karin Landerl^1^

^1^ Institute of Psychology, University of Graz, Austria

^2^ Department of Child and Adolescent Psychiatry, Psychosomatics, and Psychotherapy, Ludwig-Maximilian University, Munich, Germany

The last hypothesis focused on whether conflict-related amplitude modulation appears in the RSD group at a later time window. Late cSP was analysed on the Pz electrode. This required a new segmentation: after ICA, data was segmented within the time window of -100-2500. Only items with a correct answer between 900 and 2500 ms were considered. Artifact rejection was the same as in the previous preprocessing (gradient criteria: more than 50 μV difference between two successive data points or more than 200 μV difference in a 200 ms window; absolute amplitude criteria: amplitudes exceeding +100 or -100 μV; low activity criterion: less than 0.5 μV activity in a 100 ms window). Baseline correction was applied with baseline at -100-0. Finally, data was resegmented with -200 to 0 relative to the correct response. Since the number of valid segments were substantially lower, we included all RSD participants who had at least 20 valid segments. This resulted in the inclusion of 7 RSD participants, with a mean of 33.14 (Sd = 10.67) Conflict and 28.43 (Sd = 7.35) Non-conflict epochs.

We conducted a repeated measures ANOVA on mean amplitudes over the Pz electrode with Stimulus-type (Conflict versus Non-Conflict) as within-subject variable. Although conflict-related amplitudes were still lower than amplitudes for non-conflicting items, the ANOVA revealed no significant differences, F(1,6) = 1.476, p = 0.270, η_p_^2^ = 0.197. That is, the pattern of results was still the reverse of the pattern observed for the other groups.

In the current study, we tested for delayed but typical cSP amplitude modulation in the time window 200 ms before correct response in the RSD group. Results still showed no significant conflict-related cSP amplitude modulation, which argues against late activation. That is, such an experimental design may not elicit *automatic* activation of the phonological codes in RSD.
